# Bacterial cellulose membranes and spheres composite with poly (l-lactic acid) through *in situ *polymerization

**DOI:** 10.1186/1753-6561-8-S4-P56

**Published:** 2014-10-01

**Authors:** Ericka Cirigo y Pérez, Guilherme Colla, Luismar Marques Porto

**Affiliations:** 1 Departament of Chemical Engineering Graduate Program, Federal University of Santa Catarina, Florianópolis, Santa Catarina, 88040-900,Brazil

## Background

Since poly(L-lactic acid) (PLLA) shows great biocompatibility as well as biodegradability [1] and bacterial cellulose (BC) is a high purity biomaterial with unique structural and mechanical properties, composed of cellulose and water [2,3], both are biopolymers with great potential for biomedical applications. This work had the purpose of producing biocomposites with properties that bring together the interesting BC and PLLA attributes. With these in mind, an *in situ *polymerization in BC membranes and spheres was made by L-lactic acid polycondensation. The presence of toxic residues in the composite was avoid by not using a catalyst [4] since it was possible to solubilized lactic acid in the BC hydrogel allowing to take advantage of the auto-polymerization property of this acid [1,4]. The characterization of the composite was made by Scanning Electron Microscopy (SEM) and cell viability was evaluated by MTS [2,5].

## Methods

BC membranes and spheres were produced by culturing *Gluconacetobacter hansenii *ATCC 23769. For polymerization, commercial L-lactic acid was dehydrated at 105 °C under vacuum, followed by a three hours pre-polymerization step at 150 °C, also under vacuum [4]. When temperature decreased to 75 °C, BC membranes and BC spheres were added. Polymerization took place under vacuum, at 90 °C and agitated at 300 rpm for 48 hours. Samples were taken each 24 hours, submerged in methanol, washed in distillated water at 30 °C and 120 rpm for 24 hours and then dried at 45 °C. Microstructure was analyzed by SEM. To evaluate the cell viability, cultures of mouse fibroblasts L929 were grown in Dulbecco's Modified Eagle Medium (DMEM) in a humidified atmosphere, at 37 °C with 5 % CO_2_. Cell viability was determined using the colorimetric assay MTS [3-(4,5-dimethylthiazol-2-yl)-5-(3-carboxymethoxyphenyl)-2-(4-sulfophenyl)-2H-tetrazolium]. Cells were seeded in a concentration of 10^5 ^cell/membrane and analyzed after 24 hours. Absorbance at 490 nm was quantified in a microplate reader [2].

## Results and conclusions

Macroscopically, several properties of the composite biomaterial were observed. Compared with BC control all samples seemed more rigid, showed a white color when hydrated and a slight brightness when dried. Those properties were more evident within the 48 hours treatment. The SEM images showed evidences of integration between the BC and PLLA. MTS assays showed that there were cell adhesion on the 48 hours samples, but it also showed that there was a significant decrease of cell viability compared with the BC control, what suggests that cells do not have the same affinity for the biocomposite compared with the pure BC. Besides, shape of the samples was also an important factor since the difference in cell viability was significant between the spheres and the membranes.
